# Pharmacological Strategies and Surgical Management of Posthemorrhagic Hydrocephalus Following Germinal Matrix-Intraventricular Hemorrhage in Preterm Infants

**DOI:** 10.2174/1570159X23666240906115817

**Published:** 2024-09-06

**Authors:** Zhao Yang, Tian Tian Luo, Ya-Lan Dai, Han-Xiao Duan, Cheong-Meng Chong, Jun Tang

**Affiliations:** 1 Department of Neurosurgery, Children’s Hospital of Chongqing Medical University. National Research Center for Child Health and Disorders, Chongqing, 400014, China;; 2 Chongqing Key Laboratory of Translational Medical Research in Cognitive Development and Learning and Memory Disorders, Chongqing, 400014, China;; 3 Department of Neurobiology, Army Medical University (Third military medical university), Chongqing, 400038, China;; 4 State Key *Laboratory of Quality Research in Chinese Medicine, Institute of Chinese Medical Sciences, University of Macau, Macao, 999078, China*

**Keywords:** Posthemorrhagic hydrocephalus, germinal matrix-intraventricular hemorrhage, preterm infants, molecular mechanisms, low birth weight, gestation

## Abstract

Germinal matrix-intraventricular hemorrhage (GM-IVH) is a detrimental neurological complication that occurs in preterm infants, especially in babies born before 32 weeks of gestation and in those with a very low birth weight. GM-IVH is defined as a rupture of the immature and fragile capillaries located in the subependymal germinal matrix zone of the preterm infant brain, and it can lead to detrimental neurological sequelae such as posthemorrhagic hydrocephalus (PHH), cerebral palsy, and other cognitive impairments. PHH following GM-IVH is difficult to treat in the clinic, and no level-one strategies have been recommended to pediatric neurosurgeons. Several cellular and molecular mechanisms of PHH following GM-IVH have been studied in animal models, but no effective pharmacological strategies have been used in the clinic. Thus, a comprehensive understanding of molecular mechanisms, potential pharmacological strategies, and surgical management of PHH is urgently needed. The present review presents a synopsis of the pathogenesis, diagnosis, and cellular and molecular mechanisms of PHH following GM-IVH and explores pharmacological strategies and surgical management.

## 1 INTRODUCTION

Germinal matrix-intraventricular hemorrhage (GM-IVH) is one of the most serious disorders of preterm newborns. It is the most common cause of brain injury in preterm infants, and its detrimental sequelae, such as posthemorrhagic hydrocephalus (PHH), cerebral palsy, motor disorders, and other neurological impairments, have life-long effects on the patients and can lead to major burdens for families. The global incidence of GM-IVH ranges from 14.7-44.7% among babies born before 32 weeks of gestation, and the incidence rate increases with lower gestational ages and birth weights [1, 2]. The incidence range of GM-IVH varies by region. Siffel *et al*. conducted a recent systematic review with 98 eligible studies worldwide to evaluate the global incidence of GM-IVH grade 2-4 among extremely preterm infants [3]. The reported global incidence range of GM-IVH grade 3-4 was 5-52% in Europe, 8-22% in North America, 5-36% in Asia, and 8-13% in Oceania, and no credible data from Africa or South America. Unfortunately, the global incidence of GM-IVH grade 2 is not well documented. Liu *et al*. reported high incidence rates of 55.2% among mainland Chinese preterm births and 68.1% among babies with a very low birth weight (VLBW) of 1500 g [4]. With the development of care and techniques in the pediatric intensive care unit (PICU) and the significant strides made in obstetrics and neonatal medicine, the survival of preterm infants has significantly improved [5]. The improvement in preterm survival indicates that more babies with GM-IVH and detrimental sequelae are in urgent need of medical management. Among the sequelae, PHH is the most challenging encountered in pediatric neurosurgery. Although the findings from many preclinical studies in animal models and clinical trials in infants have been reported, no level-one management strategies have yet been recommended to pediatric neurosurgeons. In this review, we present a synopsis of the pathophysiology and potential mechanisms of PHH development following GM-IVH and integrate clinical and preclinical study findings to explore pharmacological strategies and surgical management for PHH in preterm infants with GM-IVH.

## 2 GM-IVH

### 2.1 Pathogenesis of GM-IVH

The pathogenesis of GM-IVH is complicated and multifactorial but is mostly attributed to the rupture of fragile and immature vascular vessels in the GM zone of preterm babies. The GM zone is in the subependymal region of the ventricular wall, which is abundant up to 32 weeks gestation and persists into the third trimester. The GM is richly vascularized by capillaries, but the muscle and collagen support for these capillaries is poor. Hypoxia in gestation triggers the secretion of factors of angiogenesis, such as VEGF and angiopoietin-2, which consequently results in the formation of fragile and immature capillary vessels in the GM zone. It is widely accepted that immature vessels are composed of an immature basal lamina lacking fibronectin, fewer pericytes, and deficient glial fibrillary acidic protein (GFAP) in astrocyte end-feet coverage. Moreover, these fragile and immature capillaries are very vulnerable to focal hypoxic changes and fluctuations in cerebral blood flow (CBF). The unstable cerebral autoregulation of CBF in preterm neonates results in vascular rupture. The hemorrhage may either be restricted to the GM layer or extend to the lateral ventricle and adjacent parenchymal tissue, which leads to posthemorrhagic hydrocephalus and detrimental neurological disorders. Besides gestational age and body weight at birth, there are many risk factors identified for GM-IVH, including early clamping of the umbilical cord (up to 30 seconds after birth), symptoms of intrauterine infection in the mother and the newborn, newborns with blood clotting disorders and genetic risk factors [6]. Although few clinical studies have beeen conducted so far, increasing attention has been paid to genetic factors. The described genetic factors include genetic mutation of polymorphisms, factor V Leiden gene mutation, and mutation of type IV collagen gene [7]. Recently, Kosik *et al*. revealed that GM-IVH was approximately two times less likely to occur in infants with the allele G of IGF-1R 3174G > A, single nucleotide polymorphisms of hemangioma-linked genes encoding for insulin-like growth factor 1 receptor [8].

### 2.2 Diagnosis and Classification of GM-IVH

Several grading systems for GM-IVH have been developed, but the systems proposed by Papile *et al*. in 1978 [9] and Volpe *et al*. in 2008 [10] are the most widely accepted. As illustrated in Fig. (**1**), Grade I and II subtypes of hemorrhage are defined as mild GM-IVH, while severe GM-IVH often refers to Grade III and IV subtypes. Using computed tomography scans, Papile *et al*. developed the following four-grade classification of GM-IVH based on the location and severity of hemorrhage: Grade I, the hemorrhage is confined to the GM zone; Grade II, the hemorrhage extends to the lateral ventricle, but ventricular dilatation is absent; Grade III, the hemorrhage extends to the lateral ventricle, and ventricular dilatation is present; and Grade IV, the hemorrhage extends into the surrounding parenchyma [9]. As a noninvasive, radiation-free, and reliable technique, cranial ultrasound (CrUS) has been the cornerstone for the diagnosis of GM-IVH since the late 1970s, with a specificity and sensitivity rate of 94% and 96%, respectively [11, 12]. In 2018, Volpe *et al*. developed the following similar four-grade classification based on CrUS findings: In grade I, the hemorrhage is confined to the subependymal GM; in Grade II, the hemorrhage extends to the lateral ventricle, but ventricular dilation is absent and/or the hemorrhage occupies less than 50% of the ventricle; Grade III, ventricular dilation is present and/or the hemorrhage occupies more than 50% of the ventricle; and Grade IV, the ventricular hemorrhage extends into the surrounding parenchyma. Currently, most cases of GM-IVH in preterm infants are diagnosed by cranial ultrasound (CrUS), which is routinely performed from before birth to a period after birth with closer surveillance. Generally, the highest risk of hemorrhage is within 48 h after birth, and most cases of GM-IVH are diagnosed by Day 7 [13], which guides the timing of series CrUS screening. It should be noted that GM-IVH may be progressive [14]. The degree of PHH following GM-IVH is determined by measuring the ventricular index from the falx to the lateral wall of the ventricular body and in the coronal plane at the level of the foramen of Monro. In Europe, bedside real-time cerebral ultrasound (cUS) is performed to diagnose GM-IVH, usually on Days 1, 3, 7, 14, and 28 following birth [15]. In 2020, the American Academy of Pediatrics recommended CrUS for all preterm infants born at ≤ 30 weeks or for those born at >30 weeks of gestation with significant risk factors [16]. As a technique that is superior to CrUS, magnetic resonance imaging (MRI) has the advantage of detecting white matter abnormalities and hemorrhagic and cystic lesions. In particular, MRI is being increasingly utilized for GM-IVH patients whose CrUS reveals severe abnormalities such as Grade III/IV GM-IVH with PHH, white matter injury (WMI), and periventricular leukomalacia (PVL). MRI requires infants to be sedated and may be unsuitable for unstable, severely ill neonates and preterm infants.

## 3 THEORIES OF PHH FOLLOWING GM-IVH

Although detrimental sequelae have often been related to the severe subtype of GM-IVH, recent studies have shown that any grade of GM-IVH may be associated with adverse outcomes. According to Christian *et al*., 9% of preterm babies with GM-IVH develop PHH, and the percentages in patients with Grade I-IV are 1%, 4%, 25%, and 28%, respectively. Among affected babies, communicating PHH accounts for most cases, which is believed to be due to the obliteration of arachnoid villi by blood debris and fibrosis with subsequent inflammation. An impairment of the CSF reabsorption system is the most widely accepted etiological theory for PHH. PHH is the most common detrimental sequelae of GM-IVH encountered in the clinic, and the underlying mechanisms remain to be elucidated. Generally, babies present debilitating neurological deficits when cerebroventricular expansion follows PHH, which results in mechanical compression of surrounding brain tissue [17].

Since Dr. Dandy developed the bulk flow theory over a century ago [18], theories explaining the etiology of PHH have evolved. In 2002, Egnor *et al*. described the vascular pulsations theory through the use of a mathematical model of communicating hydrocephalus, in which the arterial pulsations are weaker while the venous and capillary pulsations are stronger. The redistribution of vessel pulsations decreases the subarachnoid CSF-venous pressure gradient, leading to ventricle expansion [19]. Greitz *et al*. emphasized that CSF malabsorption is more an effect of vascular pulsatile redistribution than a causative factor of communicating hydrocephalus [20]. Another study suggested that the imbalance in interstitial fluid and CSF exchange caused by the disruption of Starling forces in the brain parenchymal microvasculature results in hydrocephalus development [21]. The theories of major pathway hydrocephalus and minor pathway hydrocephalus proposed by Dr. Oi and Dr. Di Rocco in 2006 increased our understanding of hydrocephalus pathophysiology. They defined major pathway hydrocephalus as a disruption of CSF circulation from the ventricles to the subarachnoid space. The minor pathway hydrocephalus is defined as a disruption of CSF circulation within the subarachnoid space and brain parenchyma [22]. In the series, they hypothesized that CSF dynamics develop in the theory of evolution from the immature brain, as the minor CSF pathway has a predominant effect on infantile hydrocephalus development [23]. The lack of vascular autoregulatory mechanisms in the fetal brain fails to prevent cerebral blood flow fluctuation following GM-IVH [24], which is in accordance with the theory of minor pathway hydrocephalus. It has been reported that the mortality of severe GM-IVH (grade III-IV) is approximately 44%, and almost 60% of survivors develop PHH. Among infants with PHH, 25% require surgery to install a shunt [25]. In another study, Robinson *et al*. reported that approximately 10% of any-grade GM-IVH patients and 20% of severe-grade GM-IVH patients will require permanent shunt installation [17].

The pathophysiology and potential mechanisms of PHH development following GM-IVH remain quite complex and vague, and no standard management has been promoted in the clinic. Shunt dependency is, of course, not desirable because of the detrimental complications of infection, occlusion, and displacement; nevertheless, it is the best definitive treatment for PHH in preterm infants. Additional surgical management has been reported to prevent PHH following GM-IVH in preterm infants, including drainage, irrigation, and fibrinolytic therapy (DRIFT) at an early stage, endoscopic third ventriculostomy (ETV), serial lumbar punctures, and endoscopic coagulation of the choroid plexus. Unfortunately, no absolute satisfactory achievement has been made. A noninvasive therapeutic strategy toward ameliorating PHH would be promising to improve the long-term quality of life for babies with GMH [26]. Pharmacological strategies have also been reported in clinical case reports, including the use of diuretics to reduce CSF production [27-29] and fibrinolytic agents [30]. To date, no effective pharmaceutical management has been devised to stop the development of PHH and subsequent neurological disorders in patients with GM-IVH [31]. However, laboratory studies focusing on potential mechanisms of PHH development following GM-IVH and intervention strategies have shown a rising trend [32-34].

## 4 POTENTIAL MECHANISMS OF PHH FOLLOWING GM-IVH

### 4.1 Blood Clots and Erythrocyte Lysis Products

PHH is caused by CSF circulation impairment and reabsorption imbalance in different periods after the initial GM-IVH. According to the grade of the hemorrhage, the ventricle may dilate 7-14 days following GM-IVH, which can progress slowly or rapidly [35]. Murphy *et al*. demonstrated that 30%-35% of infants show rapid progression of ventricular size over days to weeks [36]. Infants suffering any grade of GM-IVH can develop PHH and neurological disorders, including those with grades I and II GM-IVH, in whom the rates are 1% and 4%, respectively [37]. This can be due to the initial effects of blood leakage, which can be prevented only by strategies to reduce the incidence of GM-IVH. The best evidence of our ability to modify the incidence of GM-IVH comes from the clinical randomized controlled trial, the Early *versus* Late Ventricular Intervention Study (ELVIS) [38]. The lysis of red blood cells releases hemoglobin and iron into the ventricular and extracellular spaces, which have been reported to play key roles in PHH development. Lee *et al*. demonstrated an elevation of iron and hemoglobin metabolites in the CSF of preterm rabbit pups with PHH following IVH [39]. Strahle *et al*. demonstrated significant acute ventricular dilation after intraventricular injection of iron or hemoglobin into neonatal rat pups [40]. In addition, a series of studies reported that the administration of the iron chelator deferoxamine reduced brain edema and PHH following GMH induced by collagenase VII in rat pups [41, 42]. Therefore, the role of iron in PHH formation has been demonstrated, and more details of the associated mechanisms are needed in the future.

### 4.2 Inflammation, Gliosis, and Fibrosis

Previously, we performed a series of studies to address the key role of neuroinflammation in PHH following GM-IVH [43, 44]. Inflammatory markers in periventricular tissue and CSF, such as TNF-alpha, IL-6, and TGF-beta, are highly related to periventricular gliosis and fibrosis. Gliosis was first observed in cerebral cortical biopsies from children with hydrocephalus in 1990 [45]. Gliosis is characterized by reactive proliferation of microglia, astrocytes and oligodendrocytes. Deren *et al*. found a significant increase in Iba-1 (protein marker of microglia)-positive microglia and GFAP (protein marker of astrocytes)-positive astrocytes in the brain tissue of rat pups with PHH [46]. Recently, we developed a collagenase VII-induced GM-IVH model in neonatal rat pups and found that reactive gliosis played a key role in PHH after GMH in rat pups. In the same study, we first demonstrated the interaction between microglia and astrocytes around the ventricles. Second, we found that complement C3/C3aR is a potential molecular bridge of this interaction. Finally, we found that the inhibition of C3/C3aR by an antagonist (SB 290157) improved the hydrocephalus and cognitive deficits induced by GMH-IVH, indicating that C3/C3aR was a novel and potential therapeutic target for PHH after GMH-IVH [33].

Excess connective tissue deposition under conditions of inflammation following GM-IVH results in fibrosis [47], which disrupts the normal function of the surrounding subependymal structure and intraventricular wall. Among the factors that trigger fibrosis after GM-IVH, thrombin is widely studied in neonatal rodent models of GM-IVH. For example, ventricular injection of thrombin in Postnatal Day 4 (P4) neonatal rats resulted in ventricle dilation, neuronal cell apoptosis, and periventricular inflammation [48]. Several mechanisms have been addressed. Ventricular dilation by thrombin has been attributed to a disruption of vascular endothelial-cadherin, leading to CSF production *via* the activation of the protease-activated receptors-1 (PAR1) pathway [49]. In addition, PAR stimulation upregulates the mammalian target of rapamycin (mTOR) and exacerbates neuroinflammation *via* the cyclo-oxygenase 1 and 2 pathways. Lekic *et al*. demonstrated the neuroprotective effects of attenuated PHH and neurocognitive deficits by inhibiting mTOR and the cyclo-oxygenase 2 pathway [50]. As another important factor in PHH formation, TGF-β leads to the deposition of extracellular matrix (ECM) proteins and fibrous connective tissue [51]. The secretion of TGF-β by activated microglia can be induced by thrombin, and the deposited ECM proteins in the ventricular system disrupt CSF dynamics [52]. Previously, we reported an acute upregulation of TGF-β at 24 hours in a rat GMH model, and TGF-β 1 is the isoform most associated with PHH in neonates and adults. Manaenko *et al*. demonstrated the neuroprotective effects of SD208, a selective inhibitor of TGF-β1, in ameliorating long-term PHH by reducing vitronectin and GFAP-positive astrocytes in rats [53].

### 4.3 Others

Hydrocephalus is a common neurological complication in infants with GM-IVH with multiple etiologies, and its neurological outcomes are poor. Generally, hydrocephalus can be explained by an imbalance in the CSF secreted by the choroid plexus epithelium (CPE) and the CSF absorbed by the venous sinuses [54]. Despite the factors mentioned above, researchers have suggested that hydrocephalus may also be due to the accumulation of hyperosmotic substances and impaired transport of substances in the CSF. It is widely accepted that the osmotic gradient acts as a driving force of water molecular transfer from the ventricle to blood vessels across the blood-brain barrier (BBB) [55]. An increase in osmotic pressure due to hemorrhage results in more water collection in the ventricles, which in turn leads to hydrocephalus [56]. The core theory is the dysfunction of the maintenance of osmotic gradient homeostasis. In the osmotic gradient theory, many types of transporter proteins are reported in animal models but have not been applied in human studies [57]. Aquaporin (AQP), a transmembrane water transport protein, is the key protein for water transport in the CPE. Generally, CSF from the subarachnoid space flows into perivascular spaces around arteries and then the brain parenchyma through AQP on astrocytes. The process is called interstitial fluid (ISF) flow, and CSF in the parenchyma flows into the perivascular spaces of veins [58]. An upregulation of the AQP family water channel proteins has been found in several types of hydrocephalus. AQP1 is mostly expressed in the epithelium in mature tissues, and its expression increases first as an adaptive mechanism to decrease CSF osmolarity and increase the intracerebroventricular water content [59, 60]. As another toxic substance found in damaged brain tissue, amyloid-beta protein (Aβ) is associated with age-related brain disorders and is commonly found in adults with normal pressure hydrocephalus (NPH) [61, 62]. Generally, Aβ located in the choroid plexus leads to the upregulation of proinflammatory cytokines and matrix metalloproteinases (MMPs), which has been reported to damage tight junctions of the BBB and disrupt the blood-CSF barrier [63]. Nevertheless, in a premature rat kaolin-induced hydrocephalus model, the authors found low levels of Aβ accumulation. They proposed a plausible explanation for the difference in Aβ accumulation in adults and neonates: age differences are very important, and components of the Aβ degradative pathways are upregulated in immature brains [64]. However, no studies of Aβ accumulation following GM-IVH in humans or animal models have been reported.

In summary, the potential mechanisms of PHH following GM-IVH in preterm infants are likely multifactorial. Based on the uncovered mechanisms, pediatric neurosurgeons and researchers have utilized several management strategies in the clinic and attempts in the preclinic. In the following sections, we focused on two main aspects - the existing clinical management strategies for PHH in infants with GM-IVH and the potential pharmacological strategies with positive results in animal models of PHH following GM-IVH.

## 5 EXISTING CLINICAL MANAGEMENT STRATEGIES

The prevention of GM-IVH in preterm and low birth-weight infants should be the first step. Several pharmacological strategies have been reported, such as postnatal phenobarbital [65], antenatal indomethacin [66], vitamin K, and antenatal corticosteroid administration [67]. Among them, phenobarbital was proven to protect the preterm brain against hypoxic-ischemic injury in experimental models, and indomethacin should be a promising agent in reducing the incidence of IVH and, hence, PHH [31, 65]. Nevertheless, we focus on prevention strategies for PHH following GM-IVH in this review.

## 6 SURGICAL MANAGEMENT

### 6.1 Removal of Lysis Products and Fibrinolytic Therapy

Evidence shows that blood and its breakdown products persist for a period of months, which leads to neuroinflammation and the breakdown of the CSF flow pathway [35]. The removal of lysis products and fibrinolytic therapy has become the initial surgical management strategy in the clinic. Drainage, irrigation, and fibrinolytic therapy (DRIFT) were evaluated in a high-quality class I randomized clinic trial by Whitelaw *et al*. in 2007 [68]. Generally, DRIFT involves the insertion of right frontal catheters with intraventricular injection of fibrinolytic therapy agents (tissue plasminogen activator, tPA). Eight hours following tPA injection, irrigation with artificial CSF is recommended, followed by external drainage over 72 hours [14]. In the trial, in which 34 infants were assigned to the DRIFT group, and 36 were assigned to the standard therapy group, there was no significant difference between groups in the death rate or the rate of needing permanent shunt surgery. Moreover, 12 (35%) of 34 infants who received DRIFT suffered secondary IVH, compared with 3 (8%) out of 36 in the standard therapy group. The authors investigated the neurological outcomes 2 years following DRIFT and found that 11 (31%) out of 35 infants in the DRIFT group had severe cognitive dysfunction (Bayley cognitive scores < 55) compared with 19 (59%) out of 32 in the standard therapy group (adjusted OR: 0.17 [95% CI: 0.05-0.^57^]) [69]. Moreover, in a 10-year follow-up study in which 52 enrolled children were assigned to either the DRIFT group (*n =* 28) or the standard therapy group (*n =* 24), the authors found that infants in the DRIFT group received higher scores (69.3 + 30.1 SD), with a mean cognitive quotient (CQ) of 15.7 (95% confidence interval (CI)-2.9 to 34.2 point; *p =* 0.096) than infants in the standard therapy group (53.7 + 35.7 SD) [70]. Despite the higher rate of secondary IVH, infants in the DRIFT group were more likely to be alive and without severe cognitive impairment.

### 6.2 Temporary CSF Diversion

**Fig. (2):**
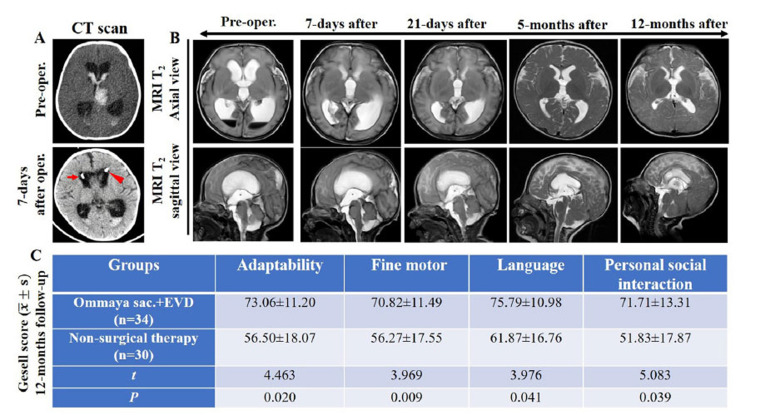
Neurological effects of EVD combined with Ommaya sac in preterm infants with PHH as primary treatment. (**A**, **B**): Images of a typical case of PHH in preterm infant before and after surgery (**A**: CT, **B**: MRI, red arrow: EVD, red arrowhead: Ommaya sac.). (**C**): Result of 12 months-Follow-up in terms of adaptability, fine motor, language and personal social interaction (unpublished data). The results were considered significant at a level of *P* < 0.05. It has since been published in the Journal of Army Medical University in Chinese as: Zou Bin, Tang Jun, Zhou Yudong, Chen Xiaobing, Zhao Xueling.: Zou Bin, Tang Jun, Zhou Yudong, Chen Xiaobing, Zhao Xueling, Liang Ping, Yu Zengpeng. Effectiveness of Ommaya sac combined with external ventricular drainage in improving neurological outcome after periventricular-intraventricular hemorrhage in neonates[J]. *J. Army Med. University*, **2022**, *44*(17): 1778-1784. DOI: 10.16016/j.2097-0927.202203222).

Surgical management of PHH following GM-IVH typically starts with temporizing lumbar punctures (LPs) for surgical evaluation [71]. As demonstrated in the ELVIS, 2-3 repeated LPs could reduce the need for permeant shunts in up to one-quarter of all patients, and when 2-3 LPs are not successful, a number of temporizing measures should be instituted (Table **1**). These include external ventricular drainage (EVD), ventriculosubgaleal shunt (VSGS) insertion, and ventricular access device (VAD) insertion. LPs are often used early in the management of PHH, although several high-quality clinical studies have reported no significant differences in the outcomes of preterm infants with PHH treated with repeated LPs or observation alone [72-74]. EVD is crucial for the drainage of blood products and for tPA injection, as reported in DRIFT, but the device cannot reside in the ventricle for a long time because of the risk of infection. The infection rate of EVD has been evaluated by many studies and ranges from unacceptable [75] to a very low level [76]. In our previous work, we combined EVD and the Ommaya Sac device at the beginning of the intervention (Fig. **2**). Generally, the EVD device was inserted into the most hemorrhagic ventricle, and the Ommaya Sac was inserted into the contralateral ventricle. Following seven days of continuous drainage with the EVD device, the Ommaya Sac device began to work at intervals, and the EVD device was removed. A total of 71 children were enrolled in the study, in which 35 infants with GM-IVH were treated with EVD and Ommaya sac insertion, and 36 infants were treated with nonsurgical therapy. An evaluation of the children’s Gesell developmental scale scores indicated that the developmental scores of the surviving children in the surgery group were significantly better than those in the nonsurgical therapy group in terms of adaptability, fine motor skills, language, and personal social interaction (*P*< 0.05) during the 12-month follow-up. A recent survey in the UK demonstrated a wide variation in practice preference for VAD (33%), VSGS (17%), ventricular puncture (25%), or repeated LPs (17%) [77]. A recent meta-analysis suggested that VAD and VSGS are equivalent in terms of the surgery risk and rate of subsequent need for permanent shunt insertion [78]. Despite the unfavorable research data, temporizing measures of CSF drainage in preterm infants with PHH are widely used strategies in the clinic. The clinical outcomes of combining different temporizing measures should be promising, but more studies are needed.

### 6.3 Permanent Shunts

Permanent ventriculoperitoneal shunt (VPS) or ventriculoatrial shunt (VAS) insertion is often performed when the temporizing measures discussed earlier fail. It has been reported that up to 18% to 39% of preterm infants who suffer from PHH after GM-IVH require permanent VPS placement. Despite the accompanying problems, such as a high rate of infection and shunt reversion, VPS is still the best definitive management strategy for PHH following GM-IVH in preterm infants. Whitelaw *et al*. suggested that a preterm infant with continuous ventricular enlargement can receive VPS placement when their body weight reaches more than 2.5 kg and their CSF protein levels are below 1.5 g/l. Controversially, a previous single-center retrospective study demonstrated no relationship between CSF glucose or protein levels, cell counts or infection, and early shunt survival [79]. A recent single-center retrospective study revealed that the rates of primary shunt failure and infection were 12.6% and 13.8%, respectively, and the infection rate of the overall shunt-related population in the same center was 8.5% [79]. The recent clinical study, BASICS, suggested a significant 3-fold reduction in shunt-related infection with the use of antibiotic-impregnated catheters compared with standard catheters [80]. In addition, for preterm infants with PHH who underwent VPS placement before the age of 1 year, 45% required shunt revision within 9 months [81]. For infants who underwent a VPS insertion that failed or who did not have adequate peritoneal reabsorption, alternative strategies included the placement of a VAS or ventriculopleural shunt and even continuing the use of a temporary shunt [17]. Due to the complications associated with VPS placement, several strategies have been studied, but no significant progress has been reported. Since VPS placement is the most effective surgical prevention strategy, research on reducing the rates of infection and revision is needed.

### 6.4 Endoscopic Third Ventriculostomy

Endoscopic third ventriculostomy (ETV) acts as an excellent alternative to permanent shunt insertion in preterm infants with obstructive hydrocephalus. However, there is not sufficient evidence to recommend the utility of ETV in preterm babies with PHH, and the studies published are all level III with an unclear degree of clinical certainty [74]. Elgamal *et al*. reviewed 52 consecutive ETV procedures and noted that preterm infants with PHH following hemorrhage did not benefit from ETV [82]. In the study, the authors reported a 77% success rate of ETV for aqueduct stenosis but a disappointing efficacy rate (14%) of ETV for the treatment of communicating hydrocephalus secondary to GM-IVH. Comfortingly, a recent pilot study of ETV combined with choroid plexus coagulation showed promise in preventing subsequent permanent shunt insertion in infants with unobstructed cisterns [83]. Therefore, ETV is a promising management strategy for further study.

### 6.5 cUS based Early Approach

Recently, more attention has focused on the cUS-based treatment strategy because of its “quiet and gentle”, especially in the critical phase of preterm babies. Moreover, functional cUS, a recently developed ultrasound technique, has been regarded as a promising technique to diagnose and predict the outcomes of interventions in preterm infants with PHVD [84]. In addition, cUS can act as a dynamic supervisor during intervention because of its relevant advantage of repeated scans to acquire real-time ventricular data during intervention. Since the percentile graphs for ventricular size has been published, supervising Ventricular Index (VI), anterior horn width (AHW), and thalamo-occipital distance (TOD) has been proven credible in predicting the outcome of PHVD treatment [84, 85]. Recently, a multicenter observational study in preterm infants with PHVD demonstrated the effectiveness of an early approach (EA) under the supervision of ventricular data with cUS [86]. In the study, 127 infants in two different neonatal units in Canada and the Netherlands were included to compare the outcomes of infants with EA or late approach (LA). Currently, EA is standard care in Dutch centers based on ventricular measurements, and infants were referred for neurosurgical intervention when ventricular measurements exceeded normal values. The interventions include LP, ventricular reservoir and even VP shunt, but LP is always the initial one. In the LA, infants were referred to neurosurgical assessment when there were signs of increased ICP. In the study, early intervention has better outcomes in PHVD infants than LA in the long term, even when eventually requiring a shunt.

## 7 PHARMACOLOGICAL STRATEGIES

### 7.1 Fibrinolytic Agents

Recently, fibrinolytic agents have been widely used in combination with ventricular drainage, despite fibrinolytic therapy being reported to be associated with a high risk of triggering secondary hemorrhage [31]. Two randomized trials have evaluated intraventricular administration of streptokinase in 12 preterm infants identified as having PHH following GM-IVH [87, 88]. No differences between the streptokinase group and the conservative group were observed in terms of the number of deaths or shunt dependence. In addition, concerns about secondary IVH and meningitis were reported, and streptokinase administered in infants before one month of age with PHH is not recommended [31]. In the DRIFT procedure, tPA was shown to be ineffective in reducing permanent shunt dependence and was associated with adverse effects [68]. There is tremendous evidence that cytokines, such as TGF-β, play a detrimental role in the development of PHH following GM-IVH [31]. Whitelaw *et al*. reported that intraventricular injection of tPA significantly increased the amount of TGF-β secreted by astrocytes, which could explain the failure of fibrinolytic agents to prevent PHH [89]. As recommended by a level I study with high clinical certainty, interventricular fibrinolytic agents, including urokinase, tPA, or streptokinase, are not recommended strategies to reduce the need for permanent shunt insertion in preterm infants with PHH [74].

### 7.2 Diuretic Therapy

In addition to fibrinolytic agents, another medical therapy is to reduce CSF production. Acetazolamide and furosemide are two clinically studied drugs administered orally or intravenously to reduce CSF production and the need for VPS placement in preterm infants. Two level I studies with high clinical certainty recommended that acetazolamide and furosemide are not effective strategies to reduce the need for permanent shunt insertion, even in patients with neurological complications [27, 90]. In the multicenter trial conducted by the International Posthemorrhagic Ventricular Dilation (PHVD) Drug Trial Group, 177 infants were treated with acetazolamide at a dose of 100 mg/kg/day and furosemide at a dose of 1 mg/kg/day. They demonstrated that the administration of acetazolamide plus furosemide resulted in a higher rate of permanent shunt insertion (relative risk 1.42) and impairments or disabilities (79% *vs.* 53%) at one year [90]. Another meta-analysis confirmed that acetazolamide and furosemide administration resulted in a borderline increase in the risk for motor impairment at one year (RR 1.27, CI 1.02 to 1.58; RD 0.16, CI 0.02 to 0.31) [28]. Regarding the mechanism of the reverse effects of diuretic therapy, a study demonstrated secondary brain injury caused by acetazolamide plus furosemide *via* cerebral vasodilatation and autoregulation dysfunction [74]. Moreover, it was found that acetazolamide may be toxic to developing oligodendrocytes [91]. Therefore, existing evidence suggests that acetazolamide and furosemide do not reduce the need for permanent VPS placement in preterm infants with PHH and are not safe.

## 8 PREVENTIVE STRATEGIES IDENTIFIED IN PRECLINICAL STUDIES

**Fig. (3):**
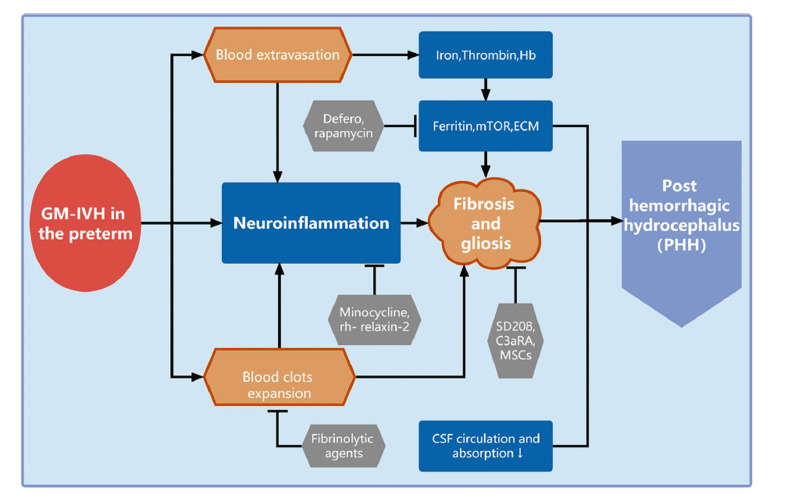
Known mechanisms and pathways in PHH development after GM-IVH and potential pharmacological treatment in preclinical studies.

Since a noninvasive therapeutic strategy toward ameliorating PHH would significantly improve the long-term quality of life for GM-IVH patients, our team has been dedicated to searching for vital molecular mechanisms as therapeutic targets for PHH following GM-IVH in preclinical studies in recent years [33, 43, 44, 92]. As there is no standard therapeutic approach toward ameliorating PHH following GM-IVH in the clinic, the existing promising potential identified in preclinical studies should be critically addressed (Fig. **3**), and further studies are needed.

### 8.1 Resolution of Blood Clot and Debris

Currently, no high-quality clinical studies have investigated the removal of blood clots in preterm infants with GM-IVH, but clinical studies involving adult hemorrhagic stroke have illustrated that surgical removal of blood clots is insufficient in improving neurological disorders. Blood clots and debris after GM-IVH disrupt normal CSF circulation and absorption and play a key role in PHH development [93]. The resolution of blood clots and debris is now a hot topic in research on ICH in adults and GM-IVH in preterm infants [93, 94]. Notably, activated microglia, induced by proinflammatory cytokines, play a detrimental role in the formation of PHH following GM-IVH [92]. However, these innate immune cell in the brain have a dual role, as M2 microglia polarize to macrophages, which are recruited to the site of injury and engulf blood clots and debris [95]. Currently, CD36 is the most investigated receptor on microglia in animal models of GM-IVH and shows promising potential in attenuating PHH [96]. The underlined molecular mechanisms involve peroxisome proliferator‐activated receptor gamma (PPAR‐γ) and nuclear factor erythroid 2‐related factor 2 (Nrf2). PPAR‐γ has been demonstrated to upregulate CD36 directly and promote M2 microglia/macrophage polarization phagocytic clearance of blood clots and debris, improving functional discovery [96]. Nrf2 is a critical transcription factor of PPAR‐γ activation in CD36 induction. Chemerin, as an adipokine, has been investigated in a P7 neonatal rate GM-IVH model. In the study, the authors found that recombinant human chemerin protein administered intraperitoneally or intranasally upregulated Nrf2 levels, reduced the inflammatory response, and attenuated subsequent PHH. Several Nrf2 agonists have been investigated in adult ICH animal models, including ankaflavin [97], dimethyl fumarate [98], and monascin [99], but have yet to be explored in GM-IVH models. Recently, Flores *et al*. demonstrated that annexin A1 is a promising therapy for hematoma resolution *via* the ERK/dusp1/CD36 pathway in a P7 rat model of GM-IVH [93]. Since blood clots and damaged debris tissue following GM-IVH are prognostic factors of subsequent neurological disorder and PHH, understanding M2 microglia/macrophage-mediated blood clot resolution after GMH‐IVH is vital for future effective nonsurgical management.

### 8.2 Anti-inflammation

Neuroinflammation is the initial pathological response following GM-IVH, and microglia, the innate immune cells in the central nervous system, are the trigger. In preterm infants with PHH following GM-IVH, increasing levels of inflammatory cytokines in CSF, including TNF-α, were detected [100]. Our previous study showed a significant increase in the inflammatory biomarkers IL-6, TNF-α and TGF-β in a P7 neonatal rat GM-IVH model [101]. In this study, we demonstrated that minocycline, a second-generation tetracycline-based molecule, attenuated neuroinflammation and reduced GM-IVH-induced lateral ventricular volume *via* cannabinoid receptor 2 (CB2R) activation. Recently, Li *et al*. found that intraperitoneal administration of recombinant human relaxin-2 (rh-relaxin-2) significantly attenuated neuroinflammation and ameliorated PHH after GM-IVH through the RXFP1/PI3K-AKT/TNFAIP3/NF-κB pathway [102]. Indeed, neuroinflammation has been associated with periventricular gliosis and arachnoid and meningeal fibrosis after GM-IVH [22, 103], and future studies of therapies targeting neuroinflammation in the management of PHH should be quite important and promising.

### 8.3 Antifibrosis and Gliosis

Fibrosis in PHH following GM-IVH is the result of excess connective tissue as a consequence of neuroinflammation [47], and the deposition of excess fibrous tissue may disrupt the normal function of parenchyma tissue and block normal CSF circulation [26]. Several factors have been reported to trigger fibrosis, and among them, thrombin and TGF-β have been well investigated. Thrombin is reported to be significantly active up to 10 days following GM-IVH in preterm infants and leads to an increase in extracellular matrix (ECM) protein proliferation associated with the upregulation of mammalian target of rapamycin (mTOR). Lekic *et al*. demonstrated that rapamycin, a selective mTOR inhibitor, significantly ameliorated long-term PHH in a neonatal rat GM-IVH model [50]. In addition, Klebe *et al*. proved that deferoxamine is a promising therapeutic strategy to improve long-term motor and cognitive function and attenuate PHH following GM-IVH in an animal model by significantly decreasing the expression of fibronectin and vitronectin [41]. TGF-β, secreted by activated microglia, leads to the production of ECM and connective tissue proteins, which stimulate fibrosis [51]. Among the TGF-β family members, TGF-β1 is the isoform most associated with PHH in preterm infants, adults, and neonates [104]. Previously, we demonstrated an elevation of TGF-β protein in the brain tissue around hematomas in a rat GM-IVH model. Manaenko *et al*. demonstrated that SD208 (a selective TGF-β1 inhibitor) administration significantly ameliorated GM-IVH-induced PHH and improved long-term cognitive and motor functions in a rat model [53]. Although the association of TGF-β with PHH development in preterm infants has been established, further studies concerning changes in CSF dynamics and efficient management of TGF-β degradation are needed.

Gliosis is characterized by reactive proliferation of microglia, astrocytes, and oligodendrocytes, which can be associated with the subarachnoid space and ventricular system, leading to hydrocephalus [105]. Previously, Deren *et al*. found a significant increase in Iba-1 (protein marker for microglia) and GFAP (protein marker for astrocytes) in the brain tissue of neonatal rats with PHH following GM-IVH. Accordingly, we demonstrated crosstalk between microglia and astrocytes in periventricular tissue in neonatal rats with PHH [33]. Moreover, we illustrated that complement C3a/C3aR is the key mediator of crosstalk and that intranasally administered purified human complement C3a is a promising therapy for alleviating long-term PHH in neonates. Given the important roles of the subarachnoid space in CSF absorption and astrocytes in BBB function as well as glymphatic CFS-interstitial fluid exchange, gliosis is a pivotal therapeutic target in the management of PHH in preterm infants. For example, mesenchymal stem cell (MSC) therapy has been reported as a new, effective, and promising strategy to decrease apoptosis and gliosis and inhibit the development of PHH in preterm infants [106].

### 8.4 Mesenchymal Stem Cell Therapy

MSC applications have been demonstrated to be efficient and safe for the treatment of acute brain injury [107] and neurodegenerative diseases [108] in preclinical and clinical studies. Studies in animal models have shown promising results of MSC treatment, but no class I clinical studies with high certainty exist. Previously, Park *et al*. conducted a series of animal studies and demonstrated that intraventricular transplantation of UCB-derived MSCs downregulated inflammatory cytokines in injured brain tissue and significantly attenuated PHH after GM-IVH in newborn rat pups [109]. In the study, the authors found that the neuroprotective effect of MSC transplantation occurred in a time-dependent manner when administered early (at 2 days) but not late (at 7 days) after the induction of severe GM-IVH. In 2018, a phase I randomized controlled trial (RCT, NCT 02274428) on extremely preterm infants conducted by Ahn *et al*. demonstrated the promising therapeutic potential of MSCs to attenuate brain injury and subsequent PHH [110]. However, there were only 9 patients in the trial, and further studies with more patients, as well as more information, is needed. Recently, a case study by Bozkaya *et al*., with a two-year follow-up, reported the neuroprotective effect of MSC treatment [106]. In the report, a male infant delivered at 27 weeks and 5 days gestation was diagnosed with grade-III GM-IVH, and MSC transplantation was decided upon. Human umbilical cord blood (UCB)-derived MSCs (1 x 10^7^/kg intraventricular and 1 x 10^7^/kg intravenous) were administered with cranial ultrasound. In the follow-up, a normal ventricle was detected by cranial MRI on the 133rd postnatal day,and on the 163rd postnatal day, he was discharged in generally good condition and with normal neurological examination findings. Thus, MSC transplantation may be a promising treatment strategy for GM-IVH in preterm. More studies are required to evaluate the benefits and harms of MSC therapy.

The most reported way to administer MSCs in GM-IVH patients or animal models is intraventricular transplantation combined with or without intravenous injection. Recently, intranasal administration of regenerative cells or products from stem cells for neonates with prenatal brain injury and hypoxic-ischemic (HI) stroke has been reported to be quite efficient in preventing secondary brain injury [111]. Ji *et al*. demonstrated that intranasal administration of human neural stem cells (hNSCs) ameliorated brain tissue loss and white matter injury in the neonatal rat model of HI [112]. They also proved that the differentiation of hNSCs maintained long-term survival and could be examined 42 days after intranasal administration. As to our acknowledgment, few studies are reported to prevent GM-IVH from secondary brain injury by intranasal delivery of MSCs or other regenerative cells, which should be a promising strategy in future research. The future research focus should include cell types and tissue sources (bone marrow, cord blood, adipose tissue, or peripheral blood) as well as methods and doses to administer (by intraventricular transplantation, intravenous, intratracheal, or intranasal) [113].

## CONCLUSION

PHH in preterm infants with GM-IVH continues to be a critical problem associated with cognitive and motor dysfunction, and there is no standard management strategy in the clinic. Temporary CSF diversion, such as through repeated LPs, EVD, and DRIFT, does not necessarily reduce the death rate or the need for permanent shunt placement, and permanent shunts are associated with high rates of shunt revision and infection. While recent research has focused largely on temporizing neurological intervention, researchers suggested that there is much to learn regarding the time and neurological impact of permanent CSF diversion [114]. The mechanisms involved in PHH development are multifactorial and widely accepted as CSF circulation disruption, including BBB dysfunction and the blocking of the arachnoid space and glymphatic CFS-interstitial fluid exchange.

The underlying molecular mechanisms include blood clot and debris deposition, neuroinflammation, fibrosis, and gliosis, among others. Although tremendous research advances have been achieved in preclinical and clinical settings in this field, more studies and clinical trials are needed, especially those relating mechanisms identified in preclinical studies to clinical practice.

## Figures and Tables

**Fig. (1):**
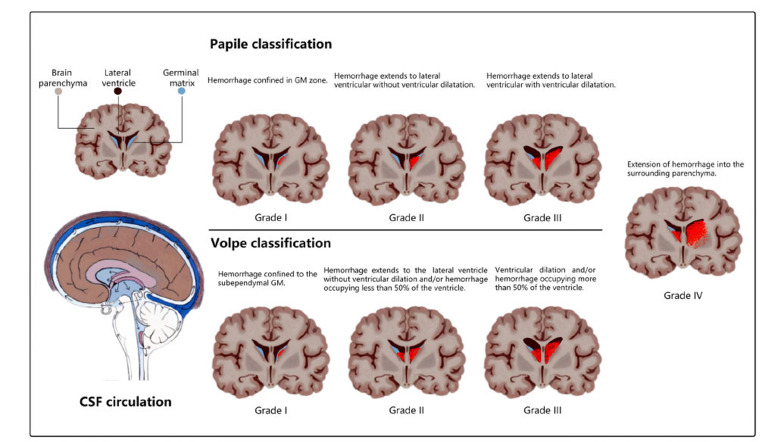
Grades of GM-IVH in papile classification and volpe classification.

**Table 1:** Literature review of several temporary CSF diversion measures.

**-**	**Authors & Year**	**Study Description**	**Results**	**Conclusions**
**Lumbar** **Puncture** **(LP)**	Anwar *et al*., 1985 [72]	Randomized controlled study of 47 preterm infants w/ PHH & Grade III or Grade IV IVH.	10 of 24 infants treated w/ LPs required shunts & 9 of 23 infants in the observation-only group required shunts.	No statistical differences in outcomes.
	Whitelaw *et al*., 2017 [115]	Two hundred eighty participants were distributed in three RCTs and one quasi-RCT. The rate of death or shunt, death or disability, and CSF infection were assessed.	Meta-analysis showed that the intervention produced no significant difference when compared to conservative management.	No evidence that repeated removal of CSF *via* LPs. Produces any benefit over conservative management in neonates.
**External** **Ventricular Drainage** **(EVD)**	Bassan *et al*., 2012 [116]	Retrospectively categorized 32 preterm infants with PHH into two groups of early (*n* = 10) or late (*n* = 22) EVD. BDI-II and neuromotor were assessed in follow-up (median: 73 months).	Early EVD *vs.* later EVD in adaptive (79 ± 22.6 *vs.* 58.8 ± 8.1, *P* = .01), personal social (90.7 ± 26 *vs.* 67.3 ± 15.9, *P =* .02), communication (95.4 ± 27.5 *vs.* 69.6 ± 20.5, *P =* .04)	Early EVD is associated with lower rates of adaptive, communication, and social disabilities. No beneficial effects in infants with prior parenchymal injury.
	Reinprecht *et al*., 2001 [117]	76 GM-IVH preterm infants enrolled. Grade II- 4, grade III -38, and grade IV -21. Early EVD applied in PHH. The rate of permanent shunt and shunt reversion was assessed in long-term follow-up to 15 years.	Forty-two infants (55.3%) need permanent shunting. Three died unrelated to the shunt. Of the surviving, 22 (56%) needed their first shunt revisions 2 days to 15 years after the first shunting procedure.	Early EVD and permanent shunting are successful methods. Infection and blockage are the main reasons for shunt revision.
**Ventricular** **Access Device (VAD)/** **Ventricular Reservoir** **(VR)**	Kormanik *et al*., 2010 [118]	Thirty-five infants with PHVD received serial reservoir taps. Ventricular reservoirs were placed in these infants for the management of hydrocephalus. The rate of infection was assessed.	13/29 (45%) infants developed blood culture-proven late-onset sepsis, but CSF cultures were negative.	VAD/VR infection neither occurred in serial taps in PHVD nor did VR infection accompany blood culture-proven sepsis. More studies are wanted.
	Christian *et al*., 2016 [37]	Retrospective review in 91 preterm infants with PHH. Fifty received temporizing VR initially, and the remaining 41 received permanent shunt, which was the first procedure. The outcome with infection shunt reversion was compared.	5/50 (10%) infants with VR as their initial procedure did not undergo subsequent VP/VA shunt placement.	No statistical difference in the number of shunt revisions and infection between the two groups. VR continues to be a safe method of temporary drainage.
**Ventriculosubgaleal Shunt (VSGs)**	Fulmer *et al*., 2000 [119]	37 VSG shunts were placed in 32 neonates. Effects and subsequent VP shunt were assessed.	Four infants died from unrelated causes of VSG shunt. Twenty-four infants received permanent VP shunt after a minimum of 4 months following VSG shunt.	VSG shunt offers a simple, effective, and relatively safe means of temporizing hydrocephalus.
	Willis *et al*. 2005 [120]	Six preterm infants with PHH underwent VSGs over one year. Clinical and imaging progress were reviewed, and the effects and complications were assessed.	1 infant (16.6%) avoided permanent VPs. 4 (66.6%) developed shunt infections.	VSGs in neonates with PHH are effective in CSF diversion. VSGs are associated with a high CSF infection rate. Studies with large case numbers are wanted.

## LIST OF ABBREVIATIONS

BBB: Blood-brain Barrier
CB2R: Cannabinoid Receptor 2
CBF: Cerebral Blood Flow
CPE: Choroid Plexus Epithelium
CQ: Cognitive Quotient
cUS: Cerebral Ultrasound
ECM: Extracellular Matrix
ETV: Endoscopic Third Ventriculostomy
EVD: External Ventricular Drainage
GFAP: Glial Fibrillary Acidic Protein
GM-IVH: Germinal Matrix-intraventricular Hemorrhage
MRI: Magnetic Resonance Imaging
MSC: Mesenchymal Stem Cell
NPH: Normal Pressure Hydrocephalus
PAR1: Protease-activated Receptors-1
PHH: Posthemorrhagic Hydrocephalus
PICU: Pediatric Intensive Care Unit
PVL: Periventricular Leukomalacia
TOD: Thalamo-occipital Distance
VLBW: Very Low Birth Weight
VSGS: Ventriculosubgaleal Shunt
